# Potential Use of CTCs as Biomarkers in Renal Cancer Patients

**DOI:** 10.3390/life12010089

**Published:** 2022-01-09

**Authors:** Joanna Bialek, Andreas Wencker, Felix Kawan, Stefan Yankulov, Paolo Fornara, Gerit Theil

**Affiliations:** Medical Faculty of Martin Luther University Halle-Wittenberg, University Clinic and Outpatient Clinic for Urology, 06120 Halle (Saale), Germany; andreas.wencker@ksa.ch (A.W.); felix.kawan@uk-halle.de (F.K.); stefan.yankulov@uk-halle.de (S.Y.); paolo.fornara@uk-halle.de (P.F.); gerit.theil@uk-halle.de (G.T.)

**Keywords:** CTC, circulating tumor cells, CellCollector, renal carcinoma, RCC, mucin, MUC1

## Abstract

We demonstrated that the CellCollector is an appropriate tool for detecting CTCs in RCC patients. We examined EpCAM and MUC1 expression levels in RCC tissues and cell lines and analyzed the detection rate of CTCs in blood samples ex vivo using an anti-EpCAM antibody-covered straight or spiraled CellCollector. Eight matched samples were examined for affinity to the anti-EpCAM vs. anti-EpCAM/anti-MUC1 antibody-covered wire. The use of this combination of antibodies allowed us to classify patients with lung metastasis. Finally, four patients were analyzed in vivo. In conclusion, both straight (ex vivo, in vivo) and spiraled (ex vivo) wires detected CTCs.

## 1. Introduction

Renal cell carcinoma (RCC) represents approximately 2% of cancer diagnoses and deaths worldwide, ranking it seventh among the most common neoplasms in industrialized countries. Patient survival depends on the timeliness of the diagnosis. At initial presentation, approximately 15% of patients already have metastases and an additional 50% may develop metastasis despite surgical resection [1,2]. As the early stage of renal cancer progression is usually asymptomatic and the first metastasis may occur before the primary tumor reaches the size range required for detection [3], there is an urgent need to evaluate methods for the early detection and monitoring of the applied therapy. Developing minimally invasive methods, such as liquid biopsy, in particular using circulating tumor cells (CTCs) as RCC markers, may help to better characterize patients, improve the classification of patients into low- or high-risk groups and monitor therapy responses in “real time”.

CTCs are cells that detach from the tumor (primary or metastatic) and enter the bloodstream, becoming circulating rare cells (CRCs) with the ability to metastasize [4]. The role of CTCs in urogenital cancers has been examined over the last ten years. Their importance was underlined in the latest guidelines (version 3) of the Prostate Cancer Clinical Trials Working Group (PCWG), in which CTC enumeration was determined to be an endpoint of clinical trials [4,5].

The CellCollector (GILUPI GmbH) is an in vivo method that uses a medical wire covered with anti-EpCAM antibodies to capture CTCs. The CellCollector is introduced into the cubital vein and within the next half hour, EpCAM-positive cells present in the bloodstream attach to the wire. Afterward, the attached cells are analyzed for cytokeratin (CK) 8, 9, 18 and 19 and CD45 expression using counter staining.

Although most RCCs originate from the epithelium, many express low levels of EpCAM or cytokeratin. The detection of intratumoral heterogeneity, which characterizes alternations in the expression pattern between tumor regions, may play a crucial role as a prognostic and predictive factor in therapy [6]. Similar situations present CTCs as a part of the liquid biopsy, which is why the isolation and characterization of CTCs may provide the opportunity to improve the statement of conventional factors. The confirmed phenotypic and molecular heterogeneity of RCC CTCs highlights the need to search for alternative or combined methods to detect these cells [7]. This can help to detect populations of epithelial (eCTC) and non-conventional (ncCTC) circulating tumor cells and is thought to increase detection [7] and therapy effectivity.

During transformation, the promotion of a malignant phenotype loosens epithelial cells and alters their polarity, consequently changing their protein expression profile. One altered protein is mucin 1 (MUC1). MUC proteins are an important element of the inflammatory response, and the deregulation of their expression could link chronic inflammation with cancer development processes [8]. The increased expression of MUC1 was found in breast [8,9], prostate [10], pancreatic [11], thyroid [12], ovarian [13] and lung [14] tumors. In RCC patients, MUC1 expression is positively associated with tumor progression [15] and nuclear grade [16], and is inversely correlated with patient survival [17].

In our pilot studies, we examined the expression of EpCAM, MUC1 and cytokeratins in renal carcinoma tissues. We tested the ability of the CellCollector to detect CTCs in RCC patient blood samples ex vivo and in vivo. To widen the detection capacity, we applied spiraled anti-EpCAM-antibody covered wire in ex vivo experiments and compared the number of captured cells with that achieved with the anti-EpCAM/MUC1-covered spiraled CellCollector.

## 2. Materials and Methods

### 2.1. Patients Enrolled in the Study

Our pilot study included a cohort (ex vivo *n* = 28; in vivo *n* = 4) of patients diagnosed with RCCs of different stages and types who were admitted to our clinic; four healthy volunteers and one patient with benign disease were enrolled as the control group (Table 1). All participants provided written informed consent. The medical faculty ethics committee of Martin Luther University Halle-Wittenberg approved the study protocol (2012–65).

### 2.2. RNA-Microarray

The total RNA was isolated from homogenized frozen tissues or primary clear cell RCC (ccRCC) cell lines (ccRCC31, ccRCC45, ccRCC122 and ccRCC162) using an RNeasy Plus MiniKit (Qiagen, Hilden, Germany) according to the manufacturer’s instructions. The microarray analysis was performed by Firma Novogene (Novogene (UK) Company Limited, Cambridge, UK).

### 2.3. Immunohistochemistry

The frozen section slides were dried for 1 h at 37 °C. After fixation with Roti-Histofix 4% (Carl Roth, Karlsruhe, Germany) for 15 min, the slides were blocked with 3% bovine serum albumin (AppliChem, Darmstadt, Germany) in PBS (Sigma/Merck, Darmstadt, Germany). Incubation with the anti-MUC1 (CellSignaling, Frankfurt am Main, Germany) antibody was performed overnight at 4 °C, followed by a 1 h incubation with the secondary rhodamine anti-mouse antibody (Dianova, Hamburg, Germany). Anti-EpCAM (Acris, Rockville, MD, USA) and anti-cytokeratin 8, 9, 18 and 19 (Abcam, Cambridge, UK) antibodies directly labeled with FITC were applied for 1 h at RT. Cell nuclei were visualized using Hoechst 33258. Images were taken using an inverted microscope at 20x magnification (Carl Zeiss Microscopy, Oberkochen, Germany).

### 2.4. Antibody Functionalization of the CellCollector System

**Figure 1:**
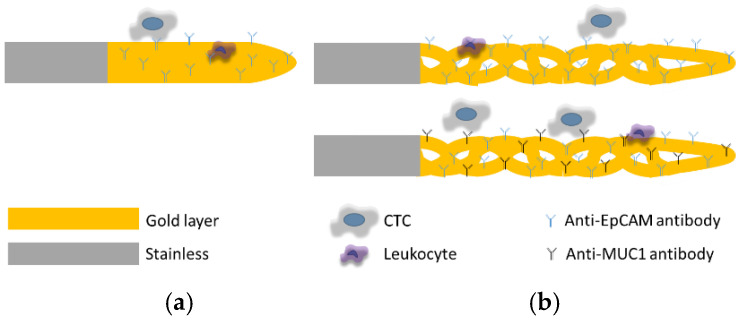
A schematic presentation of the CellCollector. Straight (**a**) and spiraled (**b**) medical wire covered with anti-EpCAM (blue) and/or anti-MUC1 (black) antibody.

Theil et al. [18] previously described the functionalization of the CellCollector. In our study, we used 16-cm long straight and spiraled medical stainless steel wires (obtained from GILUPI GmbH). The 4 cm spiraled and 2 cm straight tips of the wire were previously covered with a thick (0.2 µm) layer of gold and a polycarboxylate layer (1–5 µm). We incubated them for 15 min in sterile distilled water to rehydrate the hydrogel and then activated them in 1-ethyl-3-(3-dimethylaminopropyl) carbodiimide hydrochloride/N-hydroxysulfosuccinimide (EDC/NHS) solution (Sigma) for 20 min at 22 °C. Next, a 100 mM solution of NHS in 50 mM 2-(N-morpholino)ethanesulfonic acid (MES) buffer (Sigma), 0.5% EDC (Sigma), was added. Finally, the wire was rinsed using 5 mM acetic acid (Roth) and incubated with an anti-EpCAM or anti-EpCAM/MUC1 antibody for 1 h at 22 °C to achieve covalent bonding between the hydrogel and the antibody. To block the free carboxyl groups, the hydrogel-covered wire was incubated with 1-M ethanolamine hydrochloride (Sigma) at pH 8.5. After washing with distilled water, the wires were stored at 4 °C until use (Figure 1).

### 2.5. Cell Lines

The experiments were performed on primary renal carcinoma cell lines ccRCC31, ccRCC45, ccRCC122 and ccRCC162, which were obtained from the Institute of Immunology at Martin Luther University. All primary cells were cultivated in high-glucose DMEM (Gibco/Life Technologies Europe B.V., Bleiswijk, The Netherlands) enriched with 10% fetal calf serum (FCS) (Capricorn Scientific GmbH, Ebsdorfergrund, Germany) and MEM non-essential amino acid solution (Gibco/Life Technologies Europe B.V.) at an early passage.

### 2.6. Immunocytochemistry

All cell lines were cultured on sterile glass slides. The cells were then fixed with Roti-Histofix 4% (Carl Roth, Karlsruhe, Germany) for 15 min, washed with PBS (Sigma/Merck, Darmstadt, Germany), blocked with 5% milk (Th. Geyer, Berlin, Germany) in PBS and permeabilized with 0.01% Triton X-100 (Carl Roth). Incubation with the anti-MUC1 mouse antibody (Cell Signaling) was performed overnight at 4 °C. Direct FITC-labeled anti-EpCAM (Acris) and anti-pan cytokeratin (CK8, CK18, CK19, Abcam) antibodies, as well as the secondary anti-mouse antibody (Dianova), were applied the next day for 1 h at RT. Cell nuclei were visualized using Hoechst 33258 (Sigma, Darmstadt, Germany). Images were taken using an inverted fluorescence microscope at 20× magnification (Carl Zeiss Microscopy).

### 2.7. Capture of CTC

#### 2.7.1. Ex Vivo Experiments

Blood samples (15 mL) from patients and the control group were collected into ethylenediaminetetraacetic acid (EDTA) tubes and processed within 8 h in identical conditions. Functionalized anti-EpCAM or anti-EpCAM/MUC1 antibody-covered straight or spiraled wires were introduced into the hemodynamic system, imitating the blood flow in the body. Attached cells were fixed with acetone, blocked with 3% BSA/PBS solution for 30 min and stained with antibodies directly labeled with FITC against pan cytokeratin and CK 8, 9, 18 and 19 (Abcam). Nuclear-positive (Hoechst 33258, Sigma, Darmstadt, Germany) and CD45-negative (APC, Invitrogen) cells were considered CTCs. Images were taken using an inverted fluorescence microscope at 20x magnification (Carl Zeiss Microscopy).

#### 2.7.2. In Vivo Experiments

The CellCollector was introduced into the cubital vein with the help of a 20 G cannula. After 30 min, the medical wire was washed three times with PBS and the captured cells were fixed with acetone (10 min, RT) and blocked with 3% BSA/PBS solution for 30 min. Finally, the cells were fluorescently stained as described in the ex vivo experiment method section. Images were taken using an inverted fluorescence microscope at 20x magnification (Carl Zeiss Microscopy).

### 2.8. Statistical Analysis

Prism 9 (GraphPad Software, La Jolla, CA, USA) was used to perform the statistical analysis and to produce the figures. All data were tested for normal distribution (Shapiro–Wilk test). The data are presented as the median ± range. Due to the limited sample size, the significance of the differences between the results was not analyzed.

## 3. Results

### 3.1. Tissue Expression of Cell Surface Proteins

**Figure 2:**
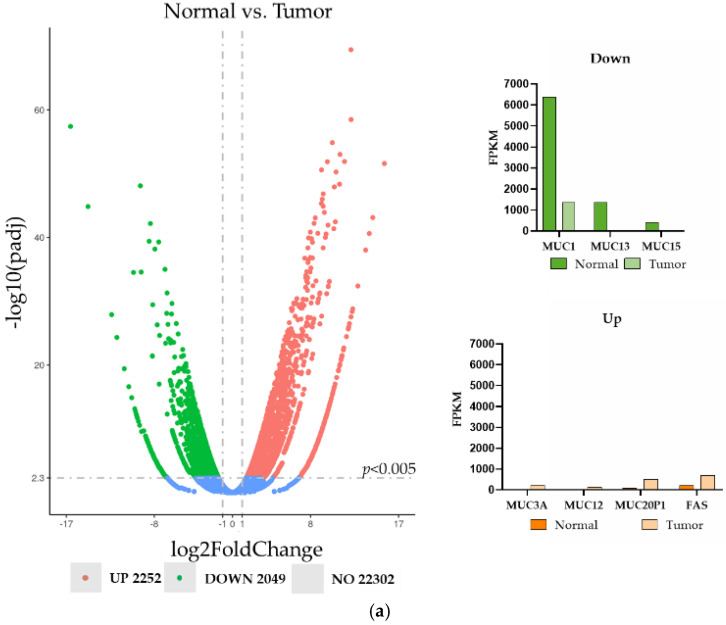

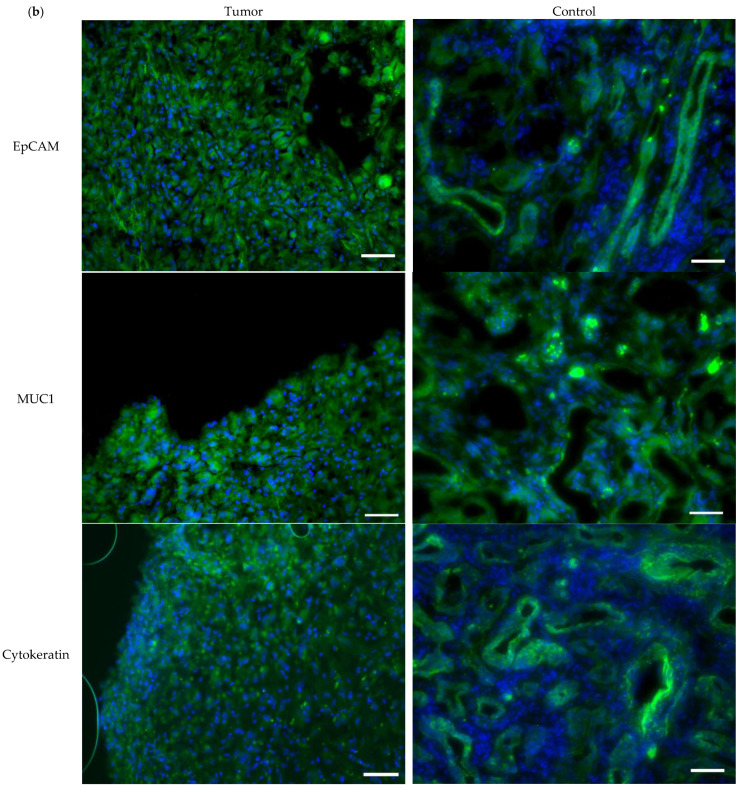
The expression analysis of the renal normal and tumor tissues. (**a**) The transcriptome microarray analysis with a detailed description of the differentially expressed surface proteins; FPKM—Fragments Per Kilobase Million; (**b**) the protein expression of EpCAM, MUC1 and cytokeratin (green) in the clear cell renal tumor (pT3b) and corresponding control tissues. The nuclei were stained with Hoechst (blue).

To analyze the expression of surface proteins as a potential candidate for CTC markers, we performed the microarray analysis on the clear cell RCC (ccRCC) tumor and corresponding control tissue. The investigations confirmed the high expression of EpCAM (not shown) and revealed the differential expression of seven surface proteins: Mucins MUC13, MUC15, MUC3A, MUC12, MUC20P1 and MUC1 and FAS (Fas cell surface death receptor). MUC13, MUC15 and MUC1 expression levels were downregulated and MUC3A, MUC12, MUC20P1 and FAS expression levels were upregulated. MUC1 and FAS displayed the most stable expression levels between the tumor and normal tissue, even though the expression level of MUC1 was higher than that of FAS (Figure 2a).

The expression of both EpCAM and MUC1, as well as cytokeratin (the standard marker for CTC detection), proteins was further tested in RCC tumors and respective controls (Figure 2b). We observed that in these diffusely structured tumors, the expression levels of EpCAM and MUC1 were higher and more homogeneous than those in the control tissues (Figure 2b).

### 3.2. Expression of EpCAM and MUC1 in Primary RCC Cell Lines

**Figure 3:**
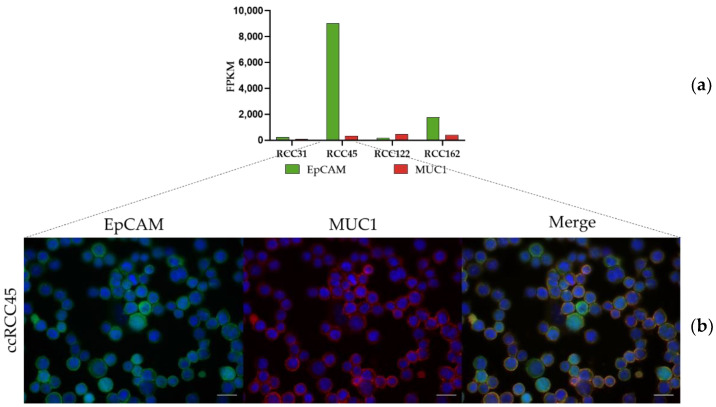
The surface markers in the primary ccRCC cell lines. (**a**) The transcriptome microarray analysis with a detailed description of EpCAM and MUC1 in ccRCC31, ccRCC45, ccRCC122 and ccRCC162; FPKM—Fragments Per Kilobase Million, (**b**) the localization of EpCAM (green) and MUC1 (red) proteins on the surface of the ccRCC45 separate cells and merged cells to visualize the colocalization. The images show protein localization. The nuclei were stained with Hoechst (blue).

Among the primary cell lines, derived from ccRCC tissues and tested with the microarray, the ccRCC45 cell line expressed the highest level of EpCAM transcripts, followed by the ccRCC162 cell line. The log2 fold change difference between the two cell lines was over two. The expression of MUC1 transcripts was comparable in three cell lines (ccRCC45, ccRCC122, ccRCC162) but low in ccRCC31 (Figure 3, Table 2).

The protein analysis confirmed the transcriptome data. The ccRCC45 cell line displayed the strongest surface staining for both EpCAM and MUC1 (Figure 3). The other cell lines demonstrated weak or very weak signals (not shown).

### 3.3. Ex Vivo EpCAM-Based Experiments Using a Hemodynamic System

During our pilot study, we performed an ex vivo analysis of the blood of RCC patients with tumors at diverse stages, as well as blood from four healthy subjects and one patient with a benign tumor as controls (Table 1). The wire type (straight or spiraled) was applied randomly. Using the anti-EpCAM antibody-covered wire, we detected CTCs in three out of six samples from the local tumor patients. The straight wires captured a median of 5.5 CTCs (range 0–11), while the spiraled wires captured a median of 2 CTCs (0–4). Together, the median number of CTCs captured from the local tumor patient samples was 2 (0–11). Three samples were negative for CTCs (Table 3). 

**Figure 4:**
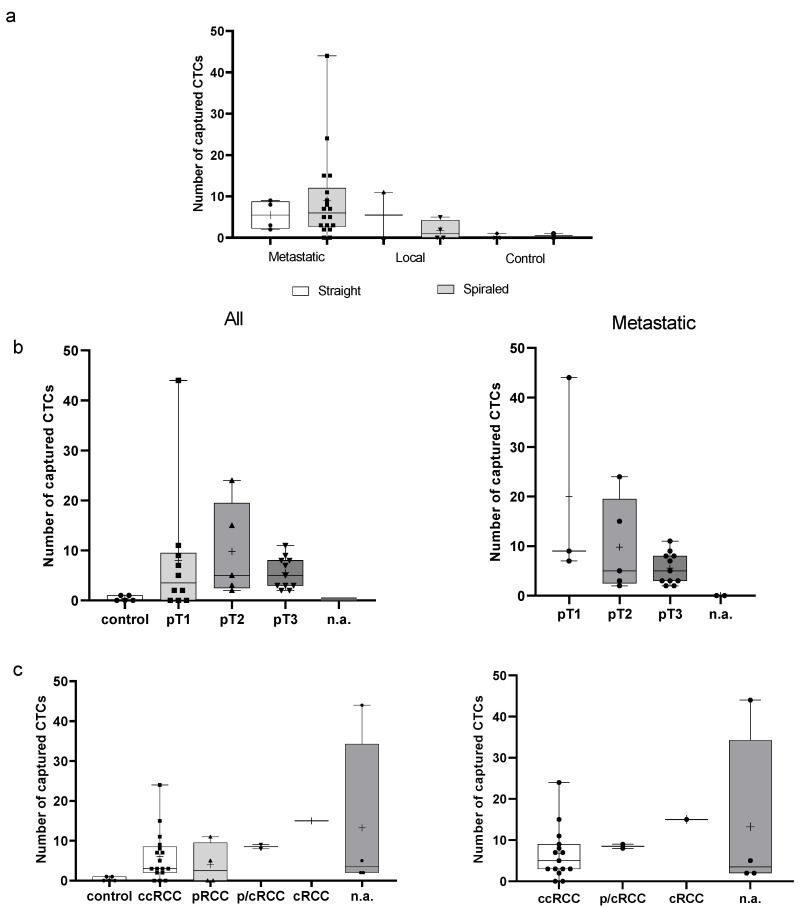
The number of CTCs captured with the CellCollector system displayed as the median and mean (“+”) with a min and max count. (**a**) Samples from the metastatic and local tumor patients with tumors at diverse stages, as well as the control group, analyzed using straight or spiraled wires. (**b**,**c**) Cells captured with both types of wire among all patient samples, or in only metastatic patient samples: (**b**) the comparison among different tumor stages; (**c**) the comparison among tumor types.

Among the 22 samples from metastatic patients, only 2 samples were negative for CTCs. The highest cell count was 44. Both the straight and spiraled wires detected a similar number of cells in the metastatic tumor patient samples, with a median (pT3) of 5.5 (range 2–9) cells obtained with the straight wire and 5 (range 2–11) with the spiraled wire. The median number of cells cached from pT1–pT3 metastatic tumor patients with the spiraled wire was 6 (range 0–44) (Table 3). In this group, we observed a decreasing trend in the median and range of captured cells (spiraled wire) as the stage of the primary tumor increased, with a median of 9 (range 7–44) in pT1 tumors, 5 (range 2–24) in pT2 tumors and 5 (range 2–11) in pT3 tumors. Two samples with missing tumor stage information were negative for CTCs (Table 3, Figure 4). 

Comparing both pT1 tumor subgroups demonstrated the difference between metastatic (median 9; range 7–44) and local (straight: median 5.5; range 0–11 and spiraled: median 2; range 0–4) tumors (Table 3, Figure 4). 

An examination of the control group revealed that only two samples (benign and healthy) were slightly positive for cells (one cell) and no cells were detected in three other samples. 

### 3.4. In Vivo EpCAM-Based Experiments

The advantage of the CellCollector is the possibility of using it in vivo, thereby omitting the blood volume limitation that exists in ex vivo investigations. The in vivo analysis was performed using a straight wire in two local and two metastatic patients. In both groups, the CellCollectors captured 2 or 10 CTCs. Both patients with 10 detected CTCs at the beginning of the therapy (first sampling) were also examined during the treatment (second sampling). The number of captured cells at this time had decreased to 4 (metastatic patient) and to 0 (local patient) (Table 3).

### 3.5. Anti-EpCAM/MUC1-Covered CellCollector

**Figure 5:**
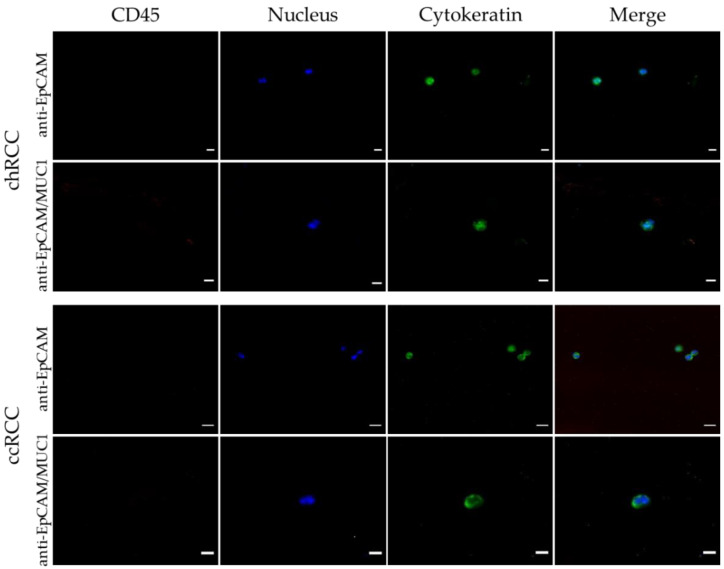
The CTCs captured by the spiraled wire. Both anti-EpCAM and anti-EpCAM/MUC1 antibody-covered CellCollector system wires were applied to the blood samples: G3 chromophiles (chRCC) and clear cells (pT3apNxpM1G2) (ccRCC). The attached cells were stained with antibodies against pan cytokeratin CK8, CK18 and CK19 (green) and CD45 (red). The nuclei were stained with Hoechst (blue).

**Figure 6:**
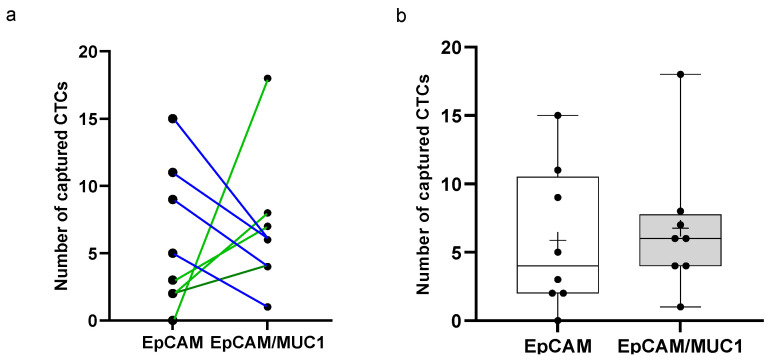
The comparison of the CellCollector wires functionalized with either anti-EpCAM or anti-EpCAM/anti-MUC1 antibodies. (**a**) The number of CTCs captured by anti-EpCAM and anti-EpCAM/anti-MUC1 antibody-covered wires in the matched paired patient samples. Green line—lung metastasis, blue line—other metastases. (**b**) The presentation of CTC detection by both wires with a median of 4 (range 0–15) for anti-EpCAM-coated wires and a median of 6 (range 1–18) for anti-EpCAM/MUC1-coated wires. The mean is displayed as “+” and the whiskers represent the min and max values.

Based on the tissues’ expression pattern, we tested whether the functionalization of the spiraled wire with two antibodies could increase the capturing capacity of the CellCollector. We examined samples from eight patients with renal tumors at diverse stages with wires covered with anti-EpCAM or anti-EpCAM/MUC1 antibodies (Figure 1 and Figure 5). The mean affinity of cells to both wire types was similar, while the median differed. The median number of cells detected by the anti-EpCAM/MUC1 antibody-covered wire was higher than that detected by the anti-EpCAM antibody-covered wire (median 6; range 1–18 vs. median 4; range 0–15) (Figure 6). The analysis of the patients’ disease history revealed that patients with higher cell numbers detected by anti-EpCAM/MUC1 than by anti-EpCAM antibody-covered wires developed pulmonary metastasis (Figure 6a).

### 3.6. Limitations

Our study was performed on blood samples from patients with different stages and subtypes of renal carcinoma. However, the study was limited by the small sample size and neither could we perform the matched comparison between straight and spiral collector. We plan to perform prospective studies with larger cohorts in future.

## 4. Discussion

There is an ongoing effort to improve the techniques of CTC detection and characterization. Most of the platforms isolating these rare cells from the bloodstream are performed ex vivo based on the physical (cell size) or biological characteristics of the cells [4]. In our studies, we showed that the CellCollector can detect renal carcinoma CTCs ex vivo and in vivo; additionally, it can be a good tool to support therapeutic decisions.

In our pilot study, we evaluated the effectiveness of the CellCollector for detecting CTCs in RCC patients. The first publication reporting the use of the CellCollector appeared in 2012 and described breast (BC) and non-small cell lung cancer (NSCLC) patients [19]. Since then, the CTCs of other tumor entities, such as lung [20], prostate [18], neuroendocrine tumors [21], squamous cell carcinoma of head and neck (SCCHN) [22] and colorectal cancer (CRC) [23], have been analyzed with this method. Comparisons with other techniques, such as CellSearch (EpCAM) or Epispot (negative depletion) [20,24], as well as specificity analyses [22,25], have confirmed the ability of the wire to capture a large number of rare tumor cells from the blood.

Despite many investigations, the tumor markers for RCC have still not been established. Tumor heterogeneity could be one of the reasons for this. The development of liquid biopsy methods has led to new opportunities to search for such markers. This less invasive diagnostic technique allows the analysis of samples from patients with different disease statuses. The examination of CTCs, in the aspect of enumeration and protein/RNA expression heterogeneity, as a part of the monitoring of tumor diseases provides information that can be used to select an appropriate therapy. One of the first clinical trials analyzing CTCs in metastatic renal cancer patients (11 patients) was performed in 2004; however, the detection rate was low [26]. The value of CTC detection as a prognostic factor and therapy response predictor in metastatic RCC patients was demonstrated in an ex vivo study by Nayak et al. [27]. By monitoring tyrosine kinase inhibitor (TKI) therapy, these researchers noticed lower progression-free survival rates in CTC-positive patients and poorer TKI therapy responses in patients with baseline CTC positivity. Recently, Kletzl et al. [28] isolated and analyzed viable renal cancer cells and examined their mitochondria, demonstrating the functional characterization of CTCs in blood samples from 186 patients undergoing surgery for RCC. In our investigation, we present the CellCollector as a tool to capture, ex vivo and in vivo, and analyze the CTCs in renal tumor patients.

The association between the frequency of CTCs and tumor aggressiveness has been demonstrated in numerous clinical trials evaluating CTCs in other tumors. An analysis of SCCHN carcinoma revealed an association between the tumor stage and the cell count range, with a wider range of CTCs in high stage tumors (0–17) than in low stage tumors (0–2) [22]. In our cohort, two low stage tumor groups existed: local and metastatic. The pathological examination of the tumors took place within a short period after surgical intervention. In the case of all pT1 metastatic patients, the analysis was performed at least 2 years before our study began; thus, at the time of the wire application, the metastatic status of the patients was confirmed. The samples from all patients diagnosed with local tumors were collected shortly before the nephrectomy was performed. The results obtained from the patients with metastases were opposite to those described for SCCHN patients (pT1: median 9; range 7–44 and pT3: median 5; range 2–11). The results obtained from the pT1 local (median 2; range 0–11) and pT3 (median 5; range 2–11) group of our cohort analyses were similar. Considering the differences in wire application time and surgical intervention, the difference between the metastatic (median 9) and local (median 2) pT1 patients obtained in our study demonstrates the importance of constant monitoring to support treatment decisions and identify disease progression. It also confirms the effectiveness of the CellCollector, as a EpCAM-based CTC capturing technique for RCC patients.

The incidence of CTCs detected in RCC patients reported in other studies is relatively low. Using subtraction enrichment and immunostaining-fluorescence in situ hybridization (SE-iFISH) technology, Tian et al. [29] detected a high number of CTCs in RCC patient samples (86.2%); nevertheless, they did not notice any significant differences between local and metastasized tumors. Using the CellSearch system, another group of investigators found CTCs only in 16% [30] or 25% [26] of analyzed blood samples. Employing the two types of CellCollector, randomly applied, we detected CTCs in 100% (straight wire) (range 2–9) and 98.82% (spiraled wire) (range 0–44) of metastatic RCC patients and 50% (straight and spiraled wire) (range 0–11) of local RCC patients ex vivo and in vivo. 

All three of the systems described above found cells in control samples. By SE-iFISH technology, the range of detected CTCs was 0–1 and the cutoff was set at 0.5 CTCs/7.5 mL [29]. When CellSearch was used, the maximal count assumed three cells and the cutoff was set at ≥2 [26]. The CellCollector captured cells in two control samples (one cell per sample). All RCC patients had more than one CTC; for this reason, detection of more than one cell could be defined as the cutoff point for the detection of RCC CTCs with the CellCollector. 

These results led us to conclude that the chosen CTC analysis method is able to detect CTCs in renal tumor patients. Using the cutoff point of more than one cell for RCCs reduces the probability of potentially false positive results.

RCC is characterized by intra- and intertumoral heterogeneity [6,31]. Even when the whole tumor area indicates a similar expression pattern, some regions may outline alternations. As reviewed by Debien et al. [32], 5% of ccRCCs and 2% of pRCCs display sarcomatoid features. According to the International Society of Urologist Pathologists, the appearance of sarcomatoid or rhabdoid components in ccRCC or pRCC tumors classify them as grade 4, which again underlines the importance of heterogeneity analysis [32].

Tumor disease development is associated with alternations, which can change the expression of epithelial markers, such as EpCAM. Tumor cells may undergo epithelial–mesenchymal transition and lose their epithelial phenotype; additionally, mesenchymal cells can transform into the epithelial phenotype [33]. Indeed, it has been demonstrated that primary tumors that are negative for EpCAM expression can yield metastases that are positive for EpCAM expression [34]. Thus, it is appropriate to recommend using EpCAM-based methods to detect RCC CTCs and to estimate the chances of progression-free survival or overall survival [35]. Our experiments demonstrated that not all primary RCC cell lines express high levels of EpCAM and such cells do not readily attach to the conventional wire. This could provide an explanation for the relatively low number of cells captured from the patient samples. 

In their preliminary report, Gradilone et al. [30] suggested the presence of renal CTCs with atypical characteristics that do not express EpCAM. The focus of their investigation was to identify a molecule that could be used to detect CTCs in RCC patients. The search for such molecules revealed several candidates; however, the lack of specificity (Carbonic Anhydrase IX) [36] or time-consuming detection protocols (VHL gene alternations in peripheral blood) [37] precludes their implementation into clinical routines.

Huang et al. [38] suggested that the protein expression profile of primary ccRCCs could influence the destination of metastatic cells, leading to preferential invasion into a particular organ. An analysis of RCC patients with pulmonary metastasis, based on the examination of CT scans performed on the date or around the time of surgery, revealed the existence of a subpopulation of cells in primary tumors that consisted of fast-growing metastatic cells. This finding correlated with the aggressive disease course [3]. Analyzing these cells could provide more information about the disease.

In our study, we found that MUC1 is a supplementary surface marker protein that can be used to detect RCC CTCs, and we propose that it can be used as an additional molecular indicator to predict the probability of lung metastasis. The overexpression of MUC1 has been found to be negatively correlated with prognoses and therapy responses in many tissues [8,13], including the kidney [16,17,39]. The expression of MUC1 induces cell invasion through the suppression of cellular aggregation and lymphocyte–target interactions, promoting the easy detachment from primary tumors and escape from the immune response [17,40,41,42]. Pulmonary metastases, the most common distant metastases of RCC, do not respond to traditional radiotherapies or chemotherapies, resulting in a poor prognosis [43]; additionally, these metastases display elevated membranous MUC1 expression levels [44]. Using the modified CellCollector wire, we detected CTCs expressing MUC1 on their surface. In one of the patient sample pairs, only the anti-mucin cover-enriched wire detected CTCs, indicating no or weak EpCAM expression. An analysis of the patients’ medical records revealed that the patients with a higher number of CTCs expressing membrane-bound MUC1 had lung metastases. There is little knowledge about expression of mucins on the surface of CTCs. Nevertheless, there is increasing evidence describing the promoting role of MUC1 in renal tumor development [16] and the elevated expression of membranous MUC1 in pulmonary metastases [44], which suggests the existence of a link combining the primary tumor, metastasis and MUC1. We propose CTC as one of the possible links. Even if we cannot confirm intra- or intertumoral heterogeneity, including the appearance of sarcomatoid or rhabdoid components, between the participants of the subsequent groups of our cohort, we cannot exclude it. The capturing of different numbers of CTCs in the matched samples by the two Collectors (EpCAM vs. EpCAM/MUC1) suggests the existence of populations of cells expressing various surface markers. This strongly supports the proposal of the heterogenic characteristics of the tumor cells. 

The importance of heterogeneity detection between primary or metastatic and circulating tumor cells is discussed by Cappelleti et al. [45]. They indicate the role of HER-2 status in benefiting the trastuzumab treatment. MUC1 plays a role in the regulation of chemoresistance in several cancer tissues, such as thyroid and breast cancers [46]. Recently, Chen et al. [39] proposed a five-gene signature (BIRC5, CD44, MUC1, TF and CCL5) to predict RCC resistance to sunitinib. The detection of MUC1 on CTCs during a relatively early metastatic stage could provide new possibilities for therapeutic intervention, as the anti-mucin 1 vaccine has already been analyzed in renal [47] and other tumor diseases [48]. Although further investigations including larger patient populations are needed, our results provide new insights into the metastatic potential of CTCs.

## 5. Conclusions

The CellCollector is able to detect CTCs in the blood of renal cancer patients. The analysis performed according to the tumor stage by the random application of either the straight or spiraled wire type demonstrated that both anti-EpCAM-antibody- and anti-EpCAM/MUC1-antibody-covered CellCollectors could capture CTCs in different types of renal carcinoma Additional functionalization with the MUC1 antibody can help to characterize patients, classify tumors as low- or high-risk and monitor therapeutic responses.

## Figures and Tables

**Table 1:** The characteristics of renal carcinoma sample donor patients and the control group.

	In Vitro		In Vivo	
Sex	*n*	%	*n*	%
Male	19	67.86	4	100
Female	9	32.14	0	0
Histology	*n*	%	*n*	%
ccRCC	14	50.00	1	25
pRCC	4	14.29	1	25
c/pRCC	1	3.57		
chRCC	1	3.57		
p.d.RCC			1	25
control	5	17.86		
n.a.	3	17.86	1	25
pT stage	*n*	%	*n*	%
pT1	10	35.71	1	25
pT2	4	14.29	2	50
pT3	8	28.57	1	25
n.a.	1	3.575		
control	5	17.86		
Wire	*n*	%	*n*	%
Smooth	8	28.57	4	100
Spiral	20	71.43		
Tumor	*n*	%	*n*	%
Local	6	19.23	2	50
Metastasis (all)	17	73.08	2	50
Bone	12	46.15 ^a^	1	25
Lung	9	34.62 ^a^	1	25
Liver	3	11.54 ^a^	1	25
Adrenal gland	4	15.38 ^a^	2	50
Lymph nodes	5	19.23 ^a^		
Other	2	7.69 ^a^		
Timepoint of wire application	*n*	%	*n*	%
>2 years before nephrectomy	14	50		
Shortly before nephrectomy	14	50	4	100
During therapy *			2	50

ccRCC—clear cell renal cell carcinoma, pRCC—papillary renal cell carcinoma, cRCC—chromophilic renal cell carcinoma, p/cRCC—papilary/chromofilic renal cell carcinoma, p.d. RCC—poorly differentiated renal cell carcinoma, n.a.—not available. ^a^ Many patients developed more than one metastasis, and the percentage shows the frequency of individual metastases among all metastatic patients. * Two patients were tested around the time of nephrectomy and during therapy.

**Table 2:** The statistically significant differential expression of EpCAM and MUC1 was determined with a microarray. All analyzed cell lines were compared with RCC45.

Cell Line	Gene Name	Log2 Fold Change	*p* Value	*p* Adj
ccRCC31	EpCAM	5.36	5.40 × 10^−13^	2.06 × 10^−11^
		5.30	1.26 × 10^−12^	4.90 × 10^−11^
ccRCC122	EpCAM	5.68	5.69 × 10^−15^	2.98 × 10^−13^
		5.61	1.59 × 10^−14^	7.83 × 10^−13^
ccRCC162	EpCAM	2.46	1.44 × 10^6^	1.34 × 10^7^
		2.51	7.69 × 10^5^	7.45 × 10^6^
ccRCC31	MUC1	1.39	0.00	0.01
		1.81	0.00	0.00

**Table 3:** The presentation of the capture of CTCs by the straight and spiraled CellCollector.

	Straight Wire—Ex Vivo		Spiraled Wire—Ex Vivo	
	Captured CTCs as Median (Range)	CTC Positive (%)	Captured CTCs as Median (Range)	CTC Positive (%)
	Metastatic			
pT1			9 (7–44)	100
pT2			5 (2–24)	100
pT3	5.5 (2–9)	100	5 (2–11)	100
n.a.			0	0
all	5.5 (2–9)	100	6 (0–44)	98.82
ccRCC	2.5 (2–3)	100	7 (0–24)	84.62
p/cRCC	8.5 (8–9)	100		
cRCC			12	100
n.a.			3.5 (2–44)	100
	Local			
pT1	5.5 (0–11)	50	2 (0–5)	50
	Control			
	0 (0–1)	33.33	0.5 (0–1)	50
	Straight Wire—In Vivo			
	Captured CTCs (range)		CTC positive (%)	
	Metastatic			
pT1	2 ^a^		100	
pT2	7 (10–4) ^b^		100	
	Local			
pT2	2 ^a^		100	
pT3	5 (10–0) ^b^		50	

ccRCC—clear cell renal cell carcinoma, pRCC—papillary renal cell carcinoma, cRCC—chromophilic renal cell carcinoma, p/cRCC—papilary/chromofilic renal cell carcinoma, p.d. RCC—poorly differentiated renal cell carcinoma, n.a.—not available. ^a^ Single wire, ^b^ data obtained from one patient displayed as the median (therapy monitoring).

## Data Availability

The data are not publicly available due to privacy and ethical restrictions.
